# Cortical‐Hypothalamic Assembloids Uncover the Cortical Regulation of Hypothalamic Responses to Fatty Acid

**DOI:** 10.1111/cpr.70207

**Published:** 2026-04-06

**Authors:** Mengdan Tao, Xiaowen Du, Qi Chen, Wenxin Mu, Shuning Lou, Xu Zhang, Min Xu, Jiting Li, Yuxuan Guo, Wanying Zhu, Yan Liu

**Affiliations:** ^1^ State Key Laboratory of Digital Medical Engineering, School of Biological Science and Medical Engineering, Department of Neurology, Affiliated Zhongda Hospital Southeast University Nanjing China; ^2^ Institute of Stem Cell and Neural Regeneration School of Pharmacy, Nanjing Medical University Nanjing China; ^3^ Institute of Cardiovascular Sciences, School of Basic Medical Sciences, State Key Laboratory of Vascular Homeostasis and Remodeling, Beijing Key Laboratory of Cardiovascular Receptors Research Peking University Beijing China

**Keywords:** assembloids, brain organoids, cortex, fatty acid, hypothalamus

## Abstract

Fatty acid (FA) overload imposes substantial stress on hypothalamic neurons, whilst whether cortical input could improve metabolic resilience of hypothalamic neurons remains poorly understood. Here, we reconstructed human cortical‐hypothalamic assembloids (CO‐HTO assembloids) to investigate how cortical input modulates hypothalamic responses to FA. Our results revealed that FA could impair neuronal survival, α‐MSH secretion, and electrophysiological activity in hypothalamic organoids (HTOs). Remarkably, fusion with cortical organoids (COs) could prevent FA‐induced apoptosis and functional defects, preserve mitochondrial respiration, and reduce lipid accumulation in HTOs. Also, transcriptomic and functional analyses revealed that cortical input could activate PGC1α‐dependent mitochondrial biogenesis. Furthermore, pharmacological PGC1α activation or glutamate treatment rescued the FA‐induced defects in HTOs. Collectively, our findings uncovered a cortico‐hypothalamic regulatory axis and found glutamate‐driven PGC1α activation might maintain hypothalamic neuronal stability and improve resilience to metabolic stress. Our CO‐HTO assembloids provided a promising platform to investigate complex inter‐regional communications and related neurological and metabolic disorders.

## Graphical abstract

By assembling cortical‐hypothalamic circuits, we show that cortical input protects hypothalamic neurons from fatty acid‐induced damage, which activates PGC1α to boost mitochondrial function. This reveals a cortico‐hypothalamic axis that defends against metabolic stress.

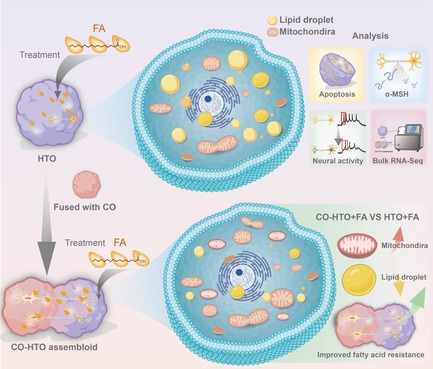

## 1 Introduction

Obesity is highly prevalent worldwide and represents major risk factor for type 2 diabetes, cardiovascular disease, and neurodegenerative disorders [1, 2]. Energy homeostasis is the core of obesity pathogenesis. This process is tightly regulated by the central nervous system (CNS), particularly the hypothalamus [3, 4]. Dysregulation of hypothalamic neuronal populations has been strongly implicated in diet‐induced obesity and metabolic dysfunction [5]. Palmitic acid (PA), the most abundant saturated fatty acid, has garnered increasing attention for its role in promoting metabolic inflammation and hypothalamic dysfunction [6, 7, 8]. However, whilst substantial progress has been made in understanding how the hypothalamus responds to lipid overload, the mechanisms by which other brain regions modulate these hypothalamic responses remain poorly defined.

The cerebral cortex serves as the centre for higher‐order cognitive and sensory processing. It anatomically and functionally interconnects with the hypothalamus through complex neural circuits [9]. Growing evidence indicates that cortical inputs may modulate hypothalamic activity in processes such as energy balance, emotion, and stress [9, 10, 11]. In addition, studies have shown that a higher body mass index (BMI) is associated with reduced overall cortical thickness [12]. Furthermore, mouse models fed a high‐fat diet exhibited decreased levels of the vesicular glutamate transporter vGlut1 in the hypothalamus [13], suggesting that the cortex may be involved in the hypothalamic response to lipid. However, how cortical regions regulate hypothalamic responses remains largely unexplored, primarily due to the lack of physiologically relevant experimental models capable of capturing interregional communication.

In recent years, brain organoids and assembloids have emerged as powerful tools to model human brain development and function in vitro [14]. Brain organoids derived from human induced pluripotent stem cells (hiPSCs) can recapitulate region‐specific neural identity, and assembloids enable the study of inter‐regional communication in a more physiologically relevant context [15, 16, 17, 18]. They enable the investigation of intricate neural processes, such as neuronal migration, long‐range circuit wiring, and neuroimmune interactions [17, 19]. Hence, assembloids have provided an unprecedented platform for studying inter‐regional communication and complex neural functions.

In this study, we developed cortical‐hypothalamic assembloids (CO‐HTO assembloids) to investigate how cortical regions modulate hypothalamic responses to FA exposure. We observed that FA‐treated HTOs exhibited a significant reduction in POMC neuronal populations and impaired neuronal electrophysiological activity. Notably, fusion with cortical organoids markedly ameliorated these FA‐induced phenotypes. Bulk RNA‐seq revealed that cortical integration was associated with a significant upregulation of mitochondrial function in the HTOs. Further mechanistic studies demonstrated that the modulation of PGC1α expression could reverse the FA‐induced hypothalamic deficits. Consistently, our data showed that exogenous glutamate treatment elevated PGC1α levels and alleviated the FA‐induced phenotypes, suggesting a potential role of cortical‐derived excitatory signalling in hypothalamic neuronal stability through PGC1α‐mediated mitochondrial support.

## 2 Materials and Methods

### 2.1 Generation of Cortical Organoids

Human induced pluripotent stem cells (hiPSCs, IMR90‐4, WiCell agreement no. 17‐W0063) were cultured in vitronectin (Thermos, A31804)‐coated 6‐well plates for 5–6 days, with E8 medium (Gibico, A1517001) maintaining their growth. To induce the differentiation of hiPSCs into cortical organoids, cell colonies were completely covered with dispase (Gibico, 17105041), then the colonies were transferred to a culture flask, where they spontaneously curled into spheres to form embryoid bodies (EBs). Neural induction medium (NIM) is prepared by adding 5 mL N2 supplement (Gibico, 17502048) and 5 mL non‐essential amino acids (NEAA, Gibico, 11,140) to 500 mL Dulbecco's Modified Eagle Medium/Nutrient Mixture F‐12 (DMEM/F12, Gibico, 11320033). From this point on, the NIM medium supplemented with SB‐431542 (2 μM, Tocris, 1614), DMH1 (2 μM, Tocris, 4126) was refreshed by half every day until day 7. On day 7, EBs were subjected to adherent culture: 10% foetal bovine serum (FBS, Gibico, 10099‐141) was added to promote EB attachment to 6‐well plates, followed by an initial 8‐h incubation. After this, EBs were continued to be cultured in NIM medium, and rosette‐containing colonies (early neural development markers) were observable under a microscope between days 10 and 14. Finally, on day 16, these rosette‐containing colonies were gently detached with a 1 mL pipette and transferred to suspension culture in NIM medium supplemented with B27 (Gibico, 12587010). From day 17 onward, the medium was changed to NIM without B27.

### 2.2 Generation of Hypothalamic Organoids

To induce the differentiation of these hiPSCs into hypothalamic organoids, approximately 1 mL of dispase was used to fully cover the cell colonies; the colonies were then transferred to a culture flask, where they spontaneously curled into spheres to form EBs. Day 0 was defined as the day when hPSCs were digested. Suspension culture was initiated on Day 0, and the induction and differentiation of thalamic organoid cells were carried out in three stages. In each stage, half of the NIM medium was replaced daily. On days 0–6, the following small molecules and factors were added to NIM medium: the Smad signalling pathway inhibitor DMH1 (1 μM), the TGF‐β receptor inhibitor A83‐01 (2 μM, StemCell Technologies, 72024), and the Wnt pathway inhibitor IWR‐1 (10 μM). Additionally, three ventralizing factors were included: SAG (1 μM, Sigma, 566660‐5MGCN), Purmorphamine (1 μM, Tocris, 455110), and Sonic hedgehog (C25II, 50 ng/mL, R&D Systems, 464‐SH). From day 7 to 12, the NIM medium was continuously supplemented with the Wnt pathway inhibitor IWR‐1 (10 μM) and the three ventralizing factors (SAG, Purmorphamine, and 20 ng/mL SHH) to sustain the induction and differentiation processes. For the long‐term culture of hypothalamic organoids from Day 13 onwards, brain‐derived neurotrophic factor (BDNF, 20 ng/mL, Gibco, 45002100UG) was used until day 20, and after day 20, the medium change could be reduced to half‐volume replacement every other day. From day 30 onward, the medium was changed to NIM supplemented with B27.

### 2.3 Generation of Striatal Organoids

HiPSCs were digested with dispase and rinsed with DMEM/F12. The cells were then transferred to culture flasks, and embryoid bodies (EBs) were formed using NIM. The medium was supplemented with SB431542 and DMH1, with half of the medium replaced daily. On day 7, the suspended EBs were plated onto culture dishes containing NIM supplemented with 10% FBS. Rosette‐containing colonies were obtained on day 10. On day 16, the differentiated rosettes were gently dislodged using a 1 mL pipette and transferred to NIM supplemented with B27 to form organoids. From day 10 to day 16, striatal induction was performed with Sonic hedgehog (C24II, R&D Systems, 1845‐SH) at a concentration of 20 ng/mL.

### 2.4 Generation of Midbrain Organoids

To generate midbrain organoids, 2 μM DMH1, 2 μM SB431542, 500 ng/mL Sonic hedgehog (C25II), and 0.4 μM CHIR99021 (Stemgent, 040004) were added to NIM. The cells were cultured in an adherent manner, with half of the medium replaced daily until day 9. Subsequently, the cells were cultured in suspension in NIM containing 2 μM SAG, 100 ng/mL SHH, and 0.4 μM CHIR99021 for 4 days. On day 13, midbrain progenitor cells were placed in NIM supplemented with 100 ng/mL FGF8b (Novoprotein, C798) and 0.5 μM SAG, and cultured for 7 days. Finally, the cells were cultured in NIM containing 20 ng/mL SHH and 10 ng/mL FGF8b for 2 weeks to obtain midbrain organoids.

### 2.5 Generation of Cortical‐Hypothalamic Assembloids

To generate cortical‐hypothalamic assembloids, we generated hypothalamic organoids and cortical organoids separately. Cortical organoids and hypothalamic organoids were selected for fusion at day 30. One organoid of each type was placed together in a U‐bottom 96‐well plate to facilitate fusion and incubated overnight in a CO_2_ incubator. The culture was maintained in NIM supplemented with B27, with half of the medium being replaced every 3–5 days.

### 2.6 Fatty Acid Treatment

PA powder (P5585, Sigma, from Guo's lab) was initially solubilised in anhydrous ethanol, yielding a primary stock solution with a concentration of 250 mM. This concentrated preparation was subsequently diluted in a solution containing 10% bovine serum albumin (BSA, product code A9418 from Sigma) to achieve a 2 mM PA working solution, which was then aliquoted and preserved at −20°C. For cell culture application, PA powder was added to the neural induction medium at a final concentration of 200 μM, followed by treatment for 48 h [20, 21].

Organoids were collected and fixed in 4% paraformaldehyde (PFA, Sigma, 158127) for 4 h, then stored at 4°C. Post‐fixation, organoids were sequentially dehydrated in 20% and 30% sucrose solutions at 4°C. Dehydration was deemed complete when organoids settled to the bottom of the container. Cryosectioning was performed using a cryotome, and sections were stored at −20°C until use. For immunofluorescence staining, tissue sections were washed three times in PBS, with each wash lasting 10 min. Sections were then blocked and permeabilized in PBS containing 5% donkey serum (Sigma, S30‐M) and 1% Triton X‐100 (Beyotime, P0096) for 60 min at room temperature. Primary antibodies were diluted in PBS containing 0.2% Triton X‐100 and 5% donkey serum, applied to sections, and incubated overnight at 4°C. Samples were kept moist and protected from light throughout incubation. The following day, sections were washed at least three times in PBS to remove excess primary antibody, then incubated with secondary antibodies (diluted in PBS containing 5% donkey serum) for 1 h at room temperature in the dark. After secondary antibody incubation, sections were washed three times in PBS.

### 2.7 Calcium Imaging

Organoids and assembloids were placed on confocal culture dishes coated with Matrigel (BD Biosciences, 356234) for calcium imaging. First, 1 μM Fluo‐4 AM (Thermos, F14201) was added to the culture dishes, followed by incubation at 37°C for 20 min. Subsequently, the organoids were left at room temperature for 20 min and then washed with DPBS (Gibco, 14190‐136). Finally, the organoids were placed in the prepared live‐cell imaging solution for imaging. Calcium imaging was performed using a confocal fluorescence microscope (Zeiss 800). The organoids were stimulated with a high‐KCL solution (67 mM). Single‐cell calcium activity analysis was conducted using ImageJ software and Prism software (v9, GraphPad). The peak [Ca^2+^] value (*F*_max_ − *F*_0_)/*F*_0_ for individual cells was calculated by dividing the difference between the maximum fluorescence intensity (*F*_max_) and the initial fluorescence intensity (F0) by the initial fluorescence intensity (F_0_).

### 2.8 Whole‐Cell Patch‐Clamp Recordings

Individual organoids at approximately 42–45 days were placed in an electrophysiological external solution containing 145 mM NaCl, 5 mM KCl, 1 mM CaCl_2_, 1 mM MgCl_2_, 5 mM HEPES, and 5 mM glucose (280 mosM, pH 7.3). Neurons within the organoids were observed under a 60× water‐immersion objective through an upright microscope (BX51WI, Olympus). Data were acquired using a MultiClamp 700B amplifier and digitised with a Digidata 1550B, followed by low‐pass filtering at 2 kHz and digitization at 20 kHz. In current‐clamp mode, neurons were held at −70 mV to induce action potentials, with a series of current steps injected (10 steps, 10 pA increments per step). Recording pipettes were fabricated from borosilicate glass using a Sutter Instrument puller (model P‐1000). These pipettes were filled with an intracellular solution containing 130 mM K‐gluconate, 10 mM KCl, 10 mM EGTA, 2 mM MgCl_2_, 0.3 mM Na‐GTP, 2 mM Na‐ATP, and 10 mM HEPES (280 mosM, pH 7.3). After immersion in the solution, the resistance of the recording pipettes should range from 7 to 11 MΩ. Voltage and current signals were acquired using an Axopatch 700B amplifier (Axon) connected to a Digidata 1322A interface (Axon), and pClamp software (version 9, Axon) was used for data processing.

### 2.9 CCK‐8 Assay

Individual organoids were cultured separately in each well of a 96‐well plate. The 10 μL of Cell Counting Kit‐8 reagent (Beyotime, C0037) was added to 100 μL of NIM in each well, followed by incubation at 37°C for 4 h. The absorbance was measured at 450 nm using a microplate reader (Infinite M Nano, TECAN) and normalised.

### 2.10 Western Blotting

Organoids and hypothalamic organoids isolated from assemblods were lysed in RIPA buffer containing protease and protease inhibitor cocktail (Sigma, 11697498001), incubated on ice for 30 min with thorough mixing, and the supernatant was collected. The protein concentration was determined using a microplate reader. Samples were then diluted to the same concentration with cell lysis buffer. After adding SDS‐PAGE protein loading buffer (Beyotime, TBSTP0015) to the samples, specific proteins were separated by electrophoresis at 120 V. Subsequently, the proteins were transferred onto a polyvinylidene fluoride membrane at 300 mA for 90 min, followed by blocking in 5% non‐fat milk at room temperature for 1 h. Primary antibodies were then added, and the membrane was incubated overnight at 4°C. The next day, the membrane was washed 5 times with TBST (Sangon Biotech, C520009‐0001) solution, 8 min each time. Secondary antibodies were incubated with the membrane on a shaker at room temperature for 2 h. After incubation, the secondary antibodies were discarded, and the membrane was washed again 5 times with TBST. Luminol substrate solutions A and B were mixed at a 1:1 volume ratio and added to the membrane surface in the dark. After 1 min, protein bands were exposed at time gradients.

### 2.11 Sample Preparation for Transmission Electron Microscope (TEM)

Brain organoids were fixed in PBS solution containing 2.5% glutaraldehyde and incubated overnight at 4°C. The next day, the organoids were washed 4 times with PBS, 15 min each time. Subsequently, 1% osmium tetroxide was added, and the samples were incubated with the cells at 4°C for 2 h. The cells were washed twice with PBS, 5 min each time. Then, 2% uranyl acetate solution was added for a 2 h incubation. Gradient dehydration was performed using 50%–100% acetone. Finally, the cells were incubated with a 1:1 volume ratio of 100% acetone and EPON812 resin for 1.5 h. The brain organoids were embedded in embedding medium overnight, followed by sectioning into ultra‐thin slices (0.05 μm). The slices were stained with uranium for 30 min and with lead for 10 min. Electron microscope images were obtained at last.

### 2.12 RNA Preparation and Real‐Time PCR

Total RNA was extracted from the organoids with a TRIzol kit (Thermo, 15,596,026), and 1 μg of total RNA from each sample was reverse transcribed into cDNA using the PrimeScrip RT reagent kit (Takara, RR037Q). The real‐time PCR was performed in a 20 μL reaction system, which included 2 μL cDNA, 1 μL forward and reverse primers, 7 μL ddH2O, and 10 μL SYBR Green RCR Master Mix (ABclonal, RX21220). The primers for qPCR used were as follows:

CD36 forward primer, 5′‐CAGGTCAACCTATTGGTCAAGCC‐3′ and reverse primer, 5′‐GCCTTCTCATCACCAATGGTCC‐3′.

FATP1 forward primer, 5′‐TGACAGTCGTCCTCCGCAAGAA‐3′ and reverse primer, 5′ CTTCAGCAGGTAGCGGCAGATC‐3′.

FATP4 forward primer, 5′‐ACGAGAGGATGATAAACTGGTGG‐3′ and reverse primer, 5′‐GCGAACTTCAGTCCAGGTCAAC‐3′.

FABP7 forward primer, 5′‐CTGTTGTTAGCCTGGATGGAGAC‐3′ and reverse primer, 5′‐CTCATAGTGGCGAACAGCAACC‐3′.

FFAR1 forward primer, 5′‐GCTGCTCTGCGTAGGACCCTA‐3′ and reverse primer, 5′‐CCAGCGGATTAAGCACCACACT‐3′.

GAPDH forward primer, 5′‐TGGAAATCCCATCACCATCTT‐3′ and reverse primer, 5′‐TGGACTCCACGACGTACT‐3′.

### 2.13 Bulk RNA‐Seq Analysis

Total RNA was extracted using the Trizol kit, and mRNA was enriched with Oligo beads, followed by transcription into cDNA. Library construction and RNA sequencing were performed on the Illumina HiSeq 2500 platform. A volcano plot was generated using the EnhancedVolcano package (v.1.10.0) to display the number of upregulated and downregulated genes. Differential RNA expression analysis was conducted with the DESeq2 package (v.1.30.0). Gene Ontology (GO) analysis and Kyoto Encyclopaedia of Genes and Genomes (KEGG) pathway analysis were performed using the clusterProfiler package (v.4.0.2). Terms with a *p*‐value < 0.05 were considered significant, and the expression of genes included in the selected terms was visualised as a heatmap using the pheatmap package (v.1.0.12). The interactions between drugs and protein targets were analysed on NetworkAnalys, a comprehensive network visualisation analysis platform based on the DrugBank database (Obio Technology).

### 2.14 Injection of Virus Into Organoids

Individual organoids were transferred to culture dishes, and 50 μL of NIM medium was added to each organoid to prevent desiccation. Viruses were removed from the −80°C freezer and immediately placed on ice. The organoids were placed under a microscope (Nikon SMZ800N). First, the location of the organoids was identified using a 4 × objective, and the magnification was adjusted according to the size of the organoids, followed by injection using a micro syringe. Each organoid was injected twice, with 0.5 μL of virus per injection. After completion of injection, 50 μL medium with B27 and penicillin–streptomycin (PS, Gibco, 2,441,849) was supplemented to each organoid. Following a 30‐min incubation, the cells were transferred to cell culture flasks, and the same medium was added.

### 2.15 Human α‐Melanocyte Stimulating Hormone (α‐MSH) ELISA Assay

The supernatant of 10 organoids was centrifuged at 1000 g for 5 min. The 100 μL of standard or supernatant was added to each well, incubated at 37°C for 2 h, and covered with the provided adhesive strip. The liquid was discarded without washing, and 100 μL of biotinylated antibody was added to each well. After covering with a new adhesive strip, the samples were incubated at 37°C for 1 h. Following liquid removal, the wells were washed three times (2 min per wash, 200 μL per well). Subsequently, 100 μL of horseradish peroxidase‐conjugated avidin was added to each well, covered with a new adhesive strip, and incubated at 37°C for 1 h. After discarding the liquid, the wells were washed five times as described previously. The 90 μL of TMB substrate was added to each well, and the samples were incubated at 37°C in the dark for 15–30 min. Next, 50 μL of stop solution was added to each well to terminate the reaction. The optical density (OD value) of each well was measured at 450 nm using a microplate reader within 5 min after reaction termination.

### 2.16 Statistical Analysis

All data were acquired from independent coverslip cultures. Offline data analysis was performed using Clampfit 10.6 (Axon), whilst statistical analysis was conducted with GraphPad Prism (v.8.0.1). Statistical significance was determined using the paired Student's *t*‐test, one‐way ANOVA, two‐way ANOVA, or nested *t*‐test. *p*‐Value < 0.05 was considered statistically significant.

### 2.17 Key Reagents and Antibodies

The key reagents and antibodies used in this study were summarised in Table S1: Key Reagents and Antibodies.

## 3 Results

### 3.1 FA Treatment Induced Neuronal Impairments in HTOs

**FIGURE 1:**
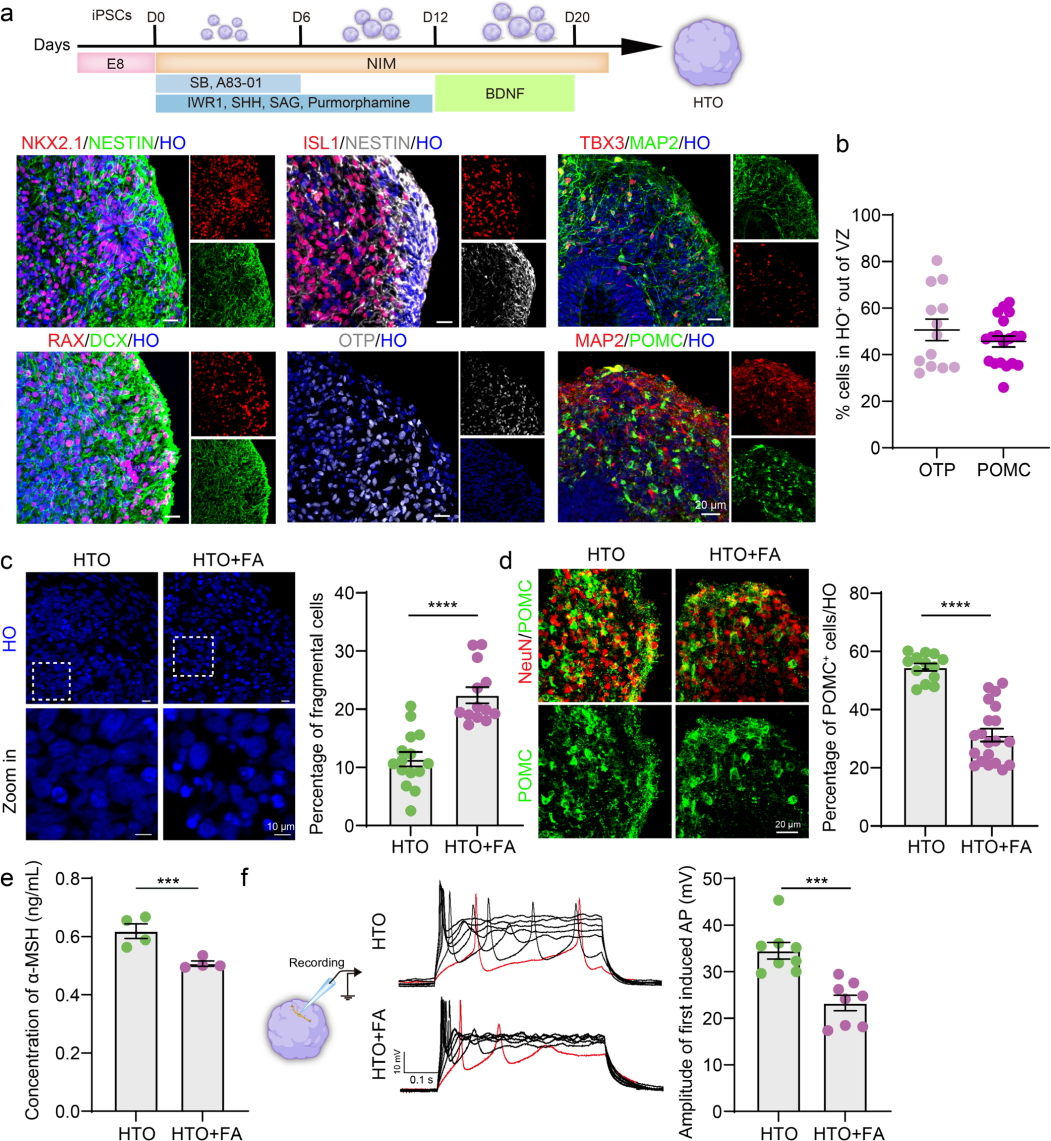
Generation and characterisation of human HTOs. (a) Schematic illustration of the protocol used to generate hypothalamic organoids from human iPSCs. Representative confocal images showing immunostaining for NKX2.1, NESTIN, TBX3, ISL1, RAX, OTP, POMC, MAP2, and Hoechst at day 30. (b) Quantification of the percentage of OTP^+^/POMC^+^ cells in HOs at day 30 (*N* ≥ 12 organoids from 3 independent experiments). (c) Representative images of fragmented cells at day 42 and quantification of their percentage (*N* ≥ 12 organoids from 3 replicates; data are presented as mean ± SEM, ****p < 0.0001). (d) Confocal images showing immunostaining for POMC and NeuN in HOs at day 42 (*N* ≥ 13 organoids from 3 replicates; mean ± SEM, ****p < 0.0001). (e) Quantification of α‐MSH levels in HOs at day 42 using ELISA (*N* = 4 biological replicates; mean ± SEM, ***p < 0.001). (f) Representative trace of neuronal action potentials and quantification of the amplitude of the first evoked AP (*N* ≥ 8 organoids from 3 replicates; mean ± SEM, ***p < 0.001).

To model the impact of lipid overload on the human hypothalamus in vitro, we first generated HTOs from human iPSCs using a differentiation protocol involving dual SMAD inhibition followed by SHH pathway activation (with SHH, SAG, and purmorphamine). At day 20 of differentiation, immunostaining analysis revealed that the organoids expressed markers of the medial ganglionic eminence (MGE) and lateral ganglionic eminence (LGE), including NKX2.1, ISL1, and the neural progenitor marker NESTIN. Also, we observed the expression of hypothalamic progenitor markers TBX3, RAX, and OTP (50.65% ± 4.64%). Early neuronal marker DCX and mature neuronal marker MAP2 were detected as well, indicating progressive neuronal differentiation. By day 40, the organoids exhibited robust expression of the hypothalamic‐specific neuronal marker POMC (45.67% ± 2.34%) (Figure 1a,b), further confirming the successful generation of a HTO model.

Then, to investigate the effects of FA on HTOs, we treated D20 organoids with FA and assessed cell proliferation and apoptosis. Quantification of KI67^+^ cells within the ventricular zone (VZ)‐like region (SOX2^+^) showed no significant difference between FA‐treated and control organoids (Figure S1). Similarly, nuclear fragmentation assays revealed no difference in apoptotic rate at D20 (Figure S1). However, after FA treatment of D40 organoids, we observed a marked increase in nuclear fragmentation (Figure 1c) accompanied by elevated Caspase‐3 expression (Figure S1), indicating enhanced apoptosis. Accordingly, we analysed the levels of CD36 and FFAR1, which are receptors of long‐chain fatty acids. We found the receptors were expressed at significantly higher levels in D40 HTOs compared with D20 HTOs (Figure S1). These differences in FA receptor expression may contribute to the increased sensitivity of D40 HTOs to FA treatment. Consistently, the proportion of POMC^+^ neurons was significantly reduced in FA‐treated organoids compared to controls (HTO: 54.58 ± 1.281%; HTO + FA: 32.24 ± 2.211%) (Figure 1d). As POMC neurons primarily release α‐MSH [22], a key hypothalamic neuropeptide, we further measured α‐MSH secretion by ELISA and found it markedly decreased after FA exposure (Figure 1e). To further assess whether FA affected neuronal activity, we performed electrophysiological and calcium imaging analyses. Patch‐clamp recordings demonstrated that FA‐treated neurons exhibited reduced action potential amplitudes (HTO: 34.50 ± 1.78 mV; HTO + FA: 23.28 ± 1.65 mV) (Figure 1f), suggesting impaired intrinsic excitability. Consistent with this, Fluo‐4 calcium imaging revealed a significant reduction in calcium transients (Figure S1). Together, these results indicate that FA treatment markedly impairs hypothalamic neuronal viability and activity.

To determine whether FA exerts similar effects on other brain regions, we differentiated iPSCs into striatum and midbrain organoids and assessed apoptosis following FA exposure. Notably, FA treatment did not induce significant cell death in either striatal or midbrain organoids (Figure S2). Furthermore, electrophysiological recordings from D40 organoids revealed no significant changes in neuronal activity after FA treatment in these two brain‐region‐specific models (Figure S2). To explore the potential basis for this regional specificity, we examined the expression of long‐chain fatty acid transporters, such as CD36. We found that the expression of CD36, FFAR1, and FATP4 was markedly higher in HTOs and mouse hypothalamic tissues compared to striatal or midbrain counterparts (Figure S2), suggesting that differential uptake of FA may underlie the region‐dependent vulnerability observed.

### 3.2 COs Alleviate Hypothalamic Cellular Abnormalities Induced by FA

**FIGURE 2:**
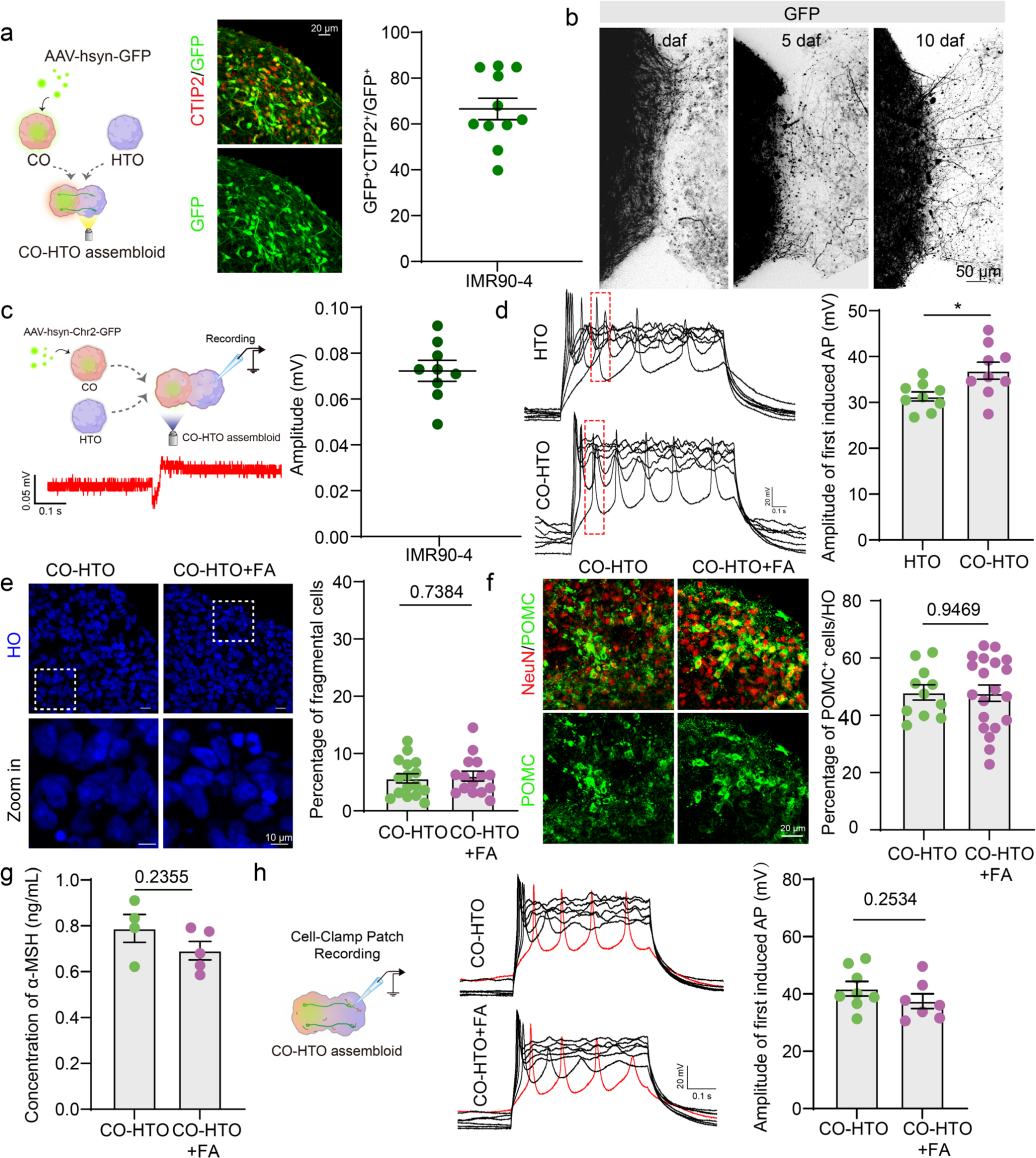
Generation of cortical–hypothalamic assembloids and functional characterisation. (a) Schematic showing the strategy for GFP infection of cortical organoids and subsequent fusion with hypothalamic organoids. Quantification of GFP^+^CTIP2^+^ cells amongst total CTIP2^+^ cells at day 30 post fusion. (b) Confocal images of GFP fluorescence at different fusion stages. (c) Representative traces of evoked field potentials recorded at 10–20 days post fusion and quantification of their amplitudes. (d) Representative traces of evoked action potentials recorded at 42–45 days post fusion and quantification of the first induced AP amplitude. (e) Representative images of fragmented cells at 12 days post fusion and quantification of their percentage (*N* ≥ 16 organoids from 3 replicates; mean ± SEM). (f) Confocal images showing POMC and NeuN immunostaining at 12 days post fusion and quantification of fragmented cells (*N* ≥ 11 organoids from 3 replicates; mean ± SEM). (g) Quantification of α‐MSH levels at 12 days post fusion by ELISA (*N* = 4 biological replicates; mean ± SEM). (h) Representative traces of neuronal action potentials and quantification of the first evoked AP amplitude (*N* ≥ 7 organoids from 3 replicates; mean ± SEM).

To investigate whether cortical input regulates the hypothalamic response to FA, we then aimed to establish a CO‐HTO assembloid model. Human iPSCs were differentiated into cortical organoids using dual‐SMAD inhibition. By day 40, the cortical organoids expressed regional and neuronal markers, including PAX6, FOXG1, CTIP2, TBR1, SATB2, DCX, TUJ1, and MAP2, confirming successful cortical specification (Figure S3). Prior to the assembly of cortical‐hypothalamic assembloids, cortical organoids were transduced with AAV‐GFP at D20 to visualise axonal projections, and efficient labelling was evident by D30 (Figure 2a). Subsequently, cortical organoids were fused with HTOs at D30 to form assembloids. Over time, the assembloids gradually increased in size, accompanied by progressive extension of GFP^+^ cortical axons towards the hypothalamic compartment (Figures 2b and S3). To determine whether functional connectivity formed between COs and HTOs, we combined optogenetics with whole‐cell patch‐clamp recording. D20 COs were first infected with AAV‐hsyn‐ChR2, and 10 days later, the COs were fused with HTOs (Figure S3). One month after fusion, blue‐light stimulation of the cortical side could elicit field potential responses in the neurons within the hypothalamic side (Figure 2c), indicating functional synaptic coupling between the two organoids. Moreover, we observed that fusion markedly increased axonal length and density of hypothalamic neurons (Figure S3). Electrophysiological recordings further revealed enhanced Na^+^ and K^+^ currents and elevated action potential amplitudes in hypothalamic neurons within assembloids (Figures 2d and S3), suggesting that cortical integration promotes hypothalamic neuronal maturation.

Next, to explore whether cortical input alters the hypothalamic response to FA, we exposed D40 CO‐HTO assembloids to FA. Notably, FA treatment did not induce nuclear fragmentation or reduce the proportion of nuclear fragmental cells and POMC^+^ neurons (Figure 2e,f), suggesting no significant apoptosis. Consistently, the level of α‐MSH, the major neuropeptide released by POMC neurons, remained unchanged after FA exposure (Figure 2g). Finally, electrophysiological and calcium imaging analyses showed no significant differences in neuronal excitability or calcium activity between FA‐treated and control assembloids (Figures 2h and S3). Together, these findings indicate that cortical‐hypothalamic connectivity confers resilience to FA‐induced dysfunction in hypothalamic neurons.

### 3.3 Bulk RNA‐Seq Reveals Changes in Mitochondrial Function‐Related Genes in HTOs After Cortical Fusion

**FIGURE 3:**
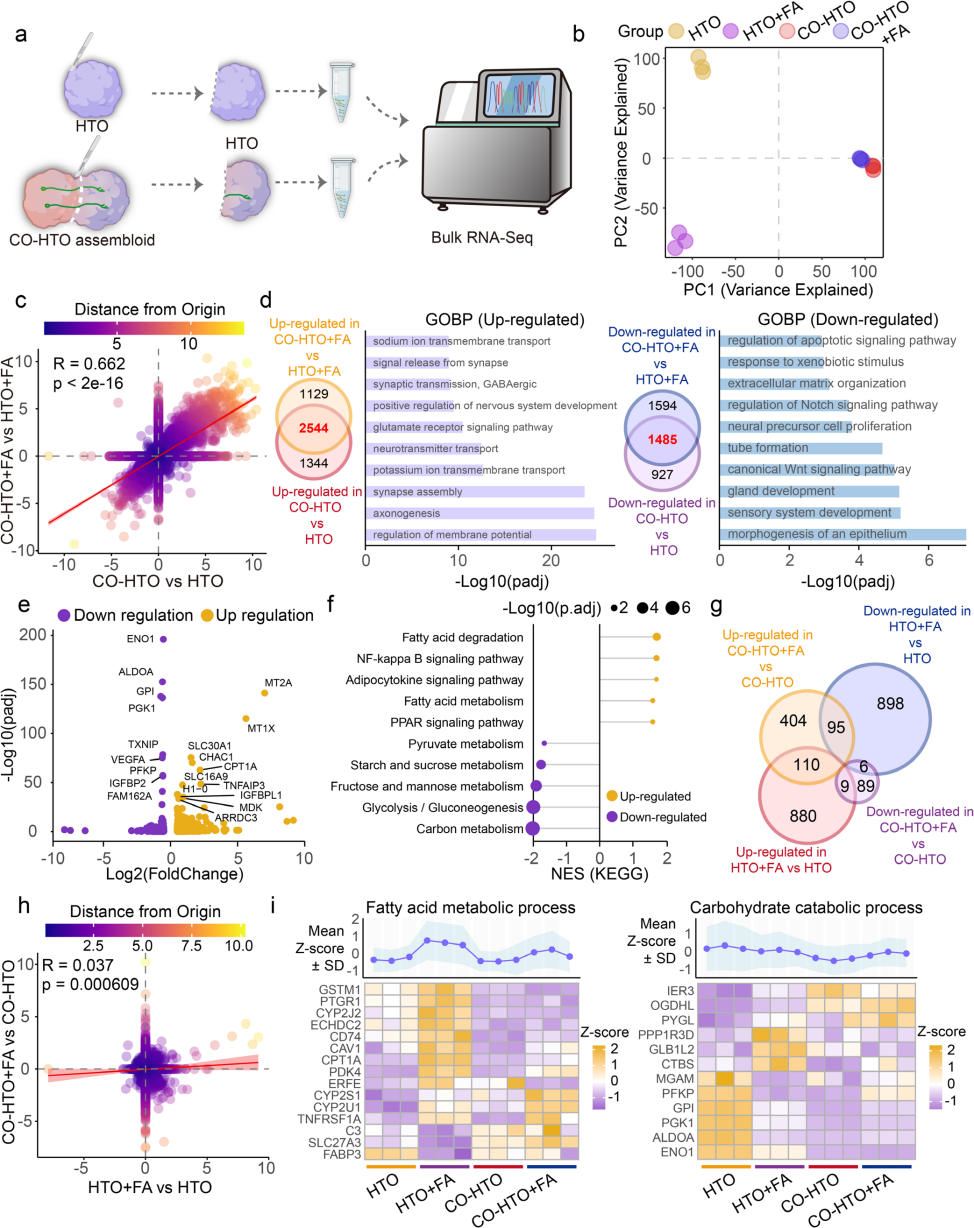
Transcriptomic profiling of hypothalamic organoids under different fatty acid exposure conditions. (a) Schematic illustration showing the preparation of bulk RNA‐seq samples from day 42 (D42) hypothalamic organoids. (b) Principal component analysis (PCA) plot showing sample clustering of HTO, HTO + FA, CO‐HTO, and CO‐HTO + FA groups. (c) Correlation analysis of differentially expressed genes between CO‐HTO + FA vs. HTO and CO‐HTO vs. HTO. (d) Venn diagrams showing the overlap of upregulated genes between CO‐HTO + FA vs. HTO + FA and CO‐HTO vs. HTO comparisons, with GO enrichment analysis of the intersected genes. Similar analyses were performed for downregulated genes. (e) Volcano plots showing altered genes between HTO + FA and HTO. (f) Pathway enrichment analysis of changed genes identified in HTO + FA vs. HTO. (g) Overlap between downregulated genes in CO‐HTO + FA vs. CO‐HTO and HTO + FA vs. HTO, and upregulated genes in HTO + FA vs. HTO and CO‐HTO + FA vs. CO‐HTO. (h) Correlation analysis of differentially expressed genes between CO‐HTO + FA vs. CO‐HTO and HTO + FA vs. HTO. (i) Heatmap showing the expression patterns of genes associated with fatty acid metabolic processes and carbohydrate catabolic processes.

To further elucidate the mechanism by which cortical‐hypothalamic connectivity confers resilience of hypothalamic neurons against FA‐induced dysfunction, we performed bulk RNA‐seq analysis. Four experimental groups were included: (1) HTOs, (2) FA‐treated HTOs (HTO + FA), (3) hypothalamic regions dissected from CO‐HTO assembloids (CO‐HTO), and (4) hypothalamic regions dissected from FA‐treated assembloids (CO‐HTO + FA). To ensure procedural consistency, non‐fused organoids were also mechanically cut once prior to RNA extraction (Figure 3a).

Principal component analysis (PCA) revealed a clear separation between HTO and HTO + FA samples, whereas the two assembloid groups (CO‐HTO and CO‐HTO + FA) clustered closely together (Figure 3b), suggesting that cortical integration mitigates FA‐induced transcriptomic alterations. Next, we compared the gene expression profiles between non‐fused and fused hypothalamic tissues under control and FA‐treated conditions. The pattern of differentially expressed genes (DEGs) between HTO vs. CO‐HTO and HTO + FA vs. CO‐HTO + FA was largely consistent (Figure 3c), indicating a shared transcriptomic signature associated with cortical fusion. Gene Ontology (GO) analysis showed that the upregulated genes in assembloids were enriched in terms related to ion channel activity, neurotransmitter transport, and synaptic signalling, whereas downregulated pathways were associated with responses to external stimuli and apoptotic processes (Figure 3d), in agreement with our previous morphological and electrophysiological findings. Volcano plot analysis further identified representative DEGs (Figure 3e). Amongst them, CPT1A, a rate‐limiting enzyme located on the outer mitochondrial membrane that facilitates long‐chain fatty acid transport into mitochondria [23], was significantly upregulated, whereas ENO1 and TXNIP, both implicated in oxidative stress and ferroptosis [24, 25], were markedly downregulated. Pathway enrichment analysis of these DEGs revealed that lipid metabolism–related pathways were strongly upregulated in the assembloids (Figure 3f), suggesting that cortical input promotes lipid resistance in hypothalamic neurons [26, 27].

To further assess how cortical organoids alter FA resilience, we compared DEGs induced by FA treatment in fused versus non‐fused conditions. Notably, the genes that were strongly dysregulated by FA in HTO exhibited attenuated or no changes in HTOs within assembloids (Figure 3g,h), indicating that cortical fusion counteracts the transcriptional impact of FA exposure. As previously reported, FA could enter the cytosol and be converted into acyl‐CoA and subsequently transported into mitochondria through CPT1 for β‐oxidation [28]. Correspondingly, analysis of mitochondrial fatty acid metabolism and carbohydrate catabolism pathways showed alterations in the assembloids after FA exposure, suggesting that cortical input helps maintain mitochondrial homeostasis under lipid overload.

Together, these data demonstrate that COs enhance the capacity of hypothalamic neurons to cope with lipid challenge, support mitochondrial stability, and thereby mitigate FA‐induced stress.

### 3.4 COs Fusion Enhanced Mitochondrial Function of HTOs Through Upregulation of PGC1α

**FIGURE 4:**
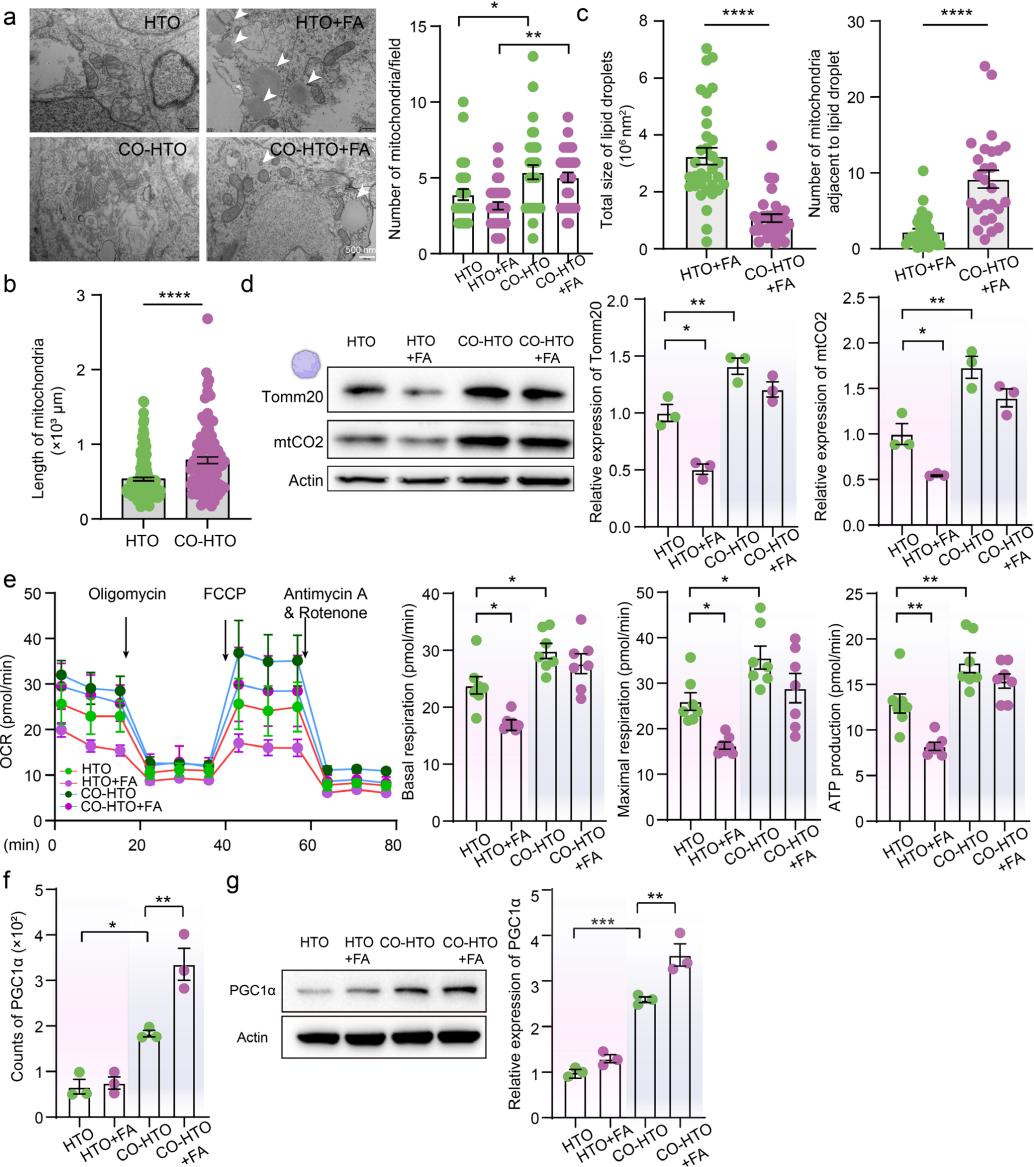
Mitochondrial dysfunction and metabolic alterations in hypothalamic organoids. (a) Representative images showing mitochondria and lipid droplets at day 42. Quantification of mitochondrial numbers across groups and the number of mitochondria surrounding lipid droplets (**p < 0.01; mean ± SEM). (b) Quantification of mitochondrial numbers across groups, and the length of mitochondria (****p < 0.0001; mean ± SEM). (c) Quantification of lipid droplet numbers across groups, and the number of mitochondria surrounding lipid droplets (****p < 0.0001; mean ± SEM). (d) Western blot analysis of TOMM20, mtCO2, and actin in different groups at D42, with quantification of relative protein expression levels (*p < 0.05, **p < 0.01; mean ± SEM). (e) Mitochondrial oxygen consumption rate (OCR) measured using a Seahorse XF96 Analyser. Quantification of basal respiration, ATP production, maximal respiration, and spare respiratory capacity (*p < 0.05, **p < 0.01; mean ± SEM). (f) Bulk RNA‐seq quantification of PGC1α expression at D42 (*p < 0.05, **p < 0.01; mean ± SEM). (g) Western blot analysis of PGC1α and actin at D42, with quantification of relative PGC1α levels (*p < 0.05, **p < 0.01; mean ± SEM).

We next examined whether cortical‐hypothalamic fusion alters mitochondrial morphology and function in hypothalamic neurons. Transmission electron microscopy (TEM) revealed a marked increase in mitochondrial abundance within HTOs after fusion (Figure 4a), indicating enhanced mitochondrial biogenesis. Meanwhile, we found the mitochondria in the CO‐HTO group were longer than those in the HTO group. (Figure 4b). Upon FA exposure, compared with the HTO group, both the number and size of lipid droplets were markedly reduced in the CO‐HTO group (Figure 4c). Notably, mitochondria clustered around lipid droplets in the CO‐HTO group were more than the HTO group, suggesting an enhanced metabolic coupling between mitochondria and lipid utilisation. These observations imply that cortical input may stimulate mitochondrial expansion and thereby enhance resistance to fatty acid overload.

To further confirm mitochondrial enrichment, we quantified mitochondrial proteins by immunoblotting (Figure 4d). The expression levels of the outer membrane protein TOMM20 and the mitochondrial complex IV subunit mtCO2 were both significantly elevated in the CO‐HTO, consistent with increased mitochondrial content. Functionally, seahorse assays demonstrated that FA treatment led to reduced oxygen consumption and ATP production, whereas after fusion with cortical organoids, the HTOs preserved mitochondrial respiration and energy output despite FA exposure (Figure 4e).

Given that PGC1α, a master regulator of mitochondrial biogenesis and energy metabolism, orchestrates the transcriptional programmes of mitochondrial biogenesis [29], we next examined its expression levels in different groups. Bulk RNA‐seq and immunoblotting analyses consistently revealed a pronounced upregulation of PGC1α in the assembloids (Figure 4f,g). These data suggested that cortical‐hypothalamic connectivity may activate a PGC1α‐driven mitochondrial biogenic programme, thereby enhancing fatty acid metabolism and conferring resilience to FA‐induced dysfunction.

Collectively, these results indicate that the fusion with cortical organoids promotes mitochondrial biogenesis and metabolic remodelling in hypothalamic neurons, which may underlie the observed protection against lipid overload.

### 3.5 PGC1α Activator and Glutamate Could Partially Rescue FA‐Induced Damage to the Hypothalamus

**FIGURE 5:**
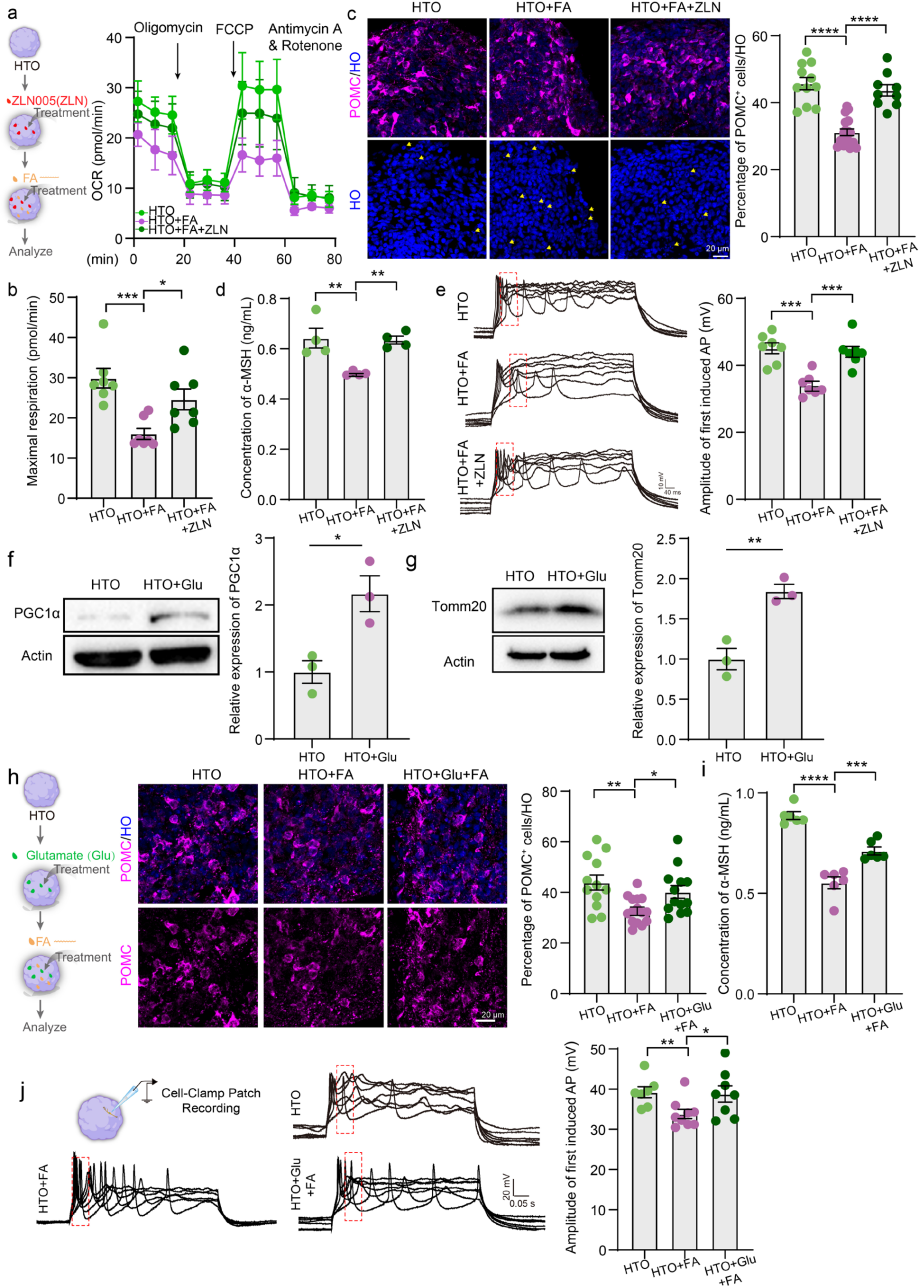
Rescue of hypothalamic organoid dysfunction by metabolic and glutamatergic interventions. (a) Mitochondrial oxygen consumption rate measured using a Seahorse XF96 Analyser. (b) Quantification of maximal respiration at D42 across treatment groups (*p < 0.05, **p < 0.01; mean ± SEM). (c) Confocal images showing POMC and HO staining in hypothalamic organoids, and quantification of POMC^+^ cells amongst total HO^+^ cells (*N* ≥ 9 organoids from 3 replicates; ***p < 0.001; mean ± SEM). (d) α‐MSH levels measured by ELISA at D42 (*N* = 4; *p < 0.05; mean ± SEM). (e) Representative traces of action potentials and quantification of the first induced AP amplitude (*N* ≥ 7 organoids from 3 replicates; **p < 0.001; mean ± SEM). (f) Western blot analysis of PGC1α and actin at D42, with quantification of relative PGC1α levels (*p < 0.05; mean ± SEM). (g) Western blot analysis of TOMM20 and actin at D42, with quantification of relative TOMM20 levels (*p* < 0.05; mean ± SEM). (h) Experimental schematic showing glutamate and palmitic acid (PA) treatment of hypothalamic organoids. Representative POMC and HO staining across groups and quantification of POMC^+^ cells amongst HO^+^ cells (*N* ≥ 14 organoids from 3 replicates; **p < 0.01, ***p < 0.001; mean ± SEM). (i) α‐MSH levels measured by ELISA at D42 (*N* = 4; ***p < 0.001, ****p < 0.0001; mean ± SEM). (j) Representative traces of action potentials and quantification of the first induced AP amplitude across groups (*N* ≥ 7 organoids from 3 replicates; *p < 0.05, **p < 0.01; mean ± SEM).

To determine whether activation of PGC1α is sufficient to mimic the protective effects conferred by cortical input, we next treated HTOs with the widely used PGC1α agonist ZLN005 (ZLN) [30, 31]. To verify the effect of ZLN005, we performed qPCR analysis, which confirmed that it significantly upregulated the expression of *PGC1α* (Figure S4). Specifically, HTOs were pretreated with 10 μM ZLN for 10 days before exposure to FA. Functional assessment of mitochondrial respiration revealed that ZLN treatment partially restored mitochondrial activity impaired by FA, as evidenced by a significant recovery of the maximal respiration rate (Figures 5a,b and S4). Consistently, quantification of apoptotic cells showed that ZLN treatment effectively reduced FA‐induced cell death (Figure S4). Furthermore, the proportion of POMC^+^ neurons and the neuropeptide α‐MSH were both rescued by ZLN administration (Figure 5c,d). Electrophysiological recordings also demonstrated that ZLN treatment restored the amplitudes of evoked action potentials, and calcium imaging confirmed the recovery of neuronal calcium transients (Figures 5e and S4). Together, these findings suggest that pharmacological activation of PGC1α ameliorates FA‐induced mitochondrial and neuronal dysfunction in HTOs.

In parallel, to investigate whether cortical‐derived excitatory neurotransmission contributes to this regulatory process, we applied glutamate to mimic cortical input. Based on reported physiological extracellular levels in the human cortex (6–7 μM) [32], and confirmed by CCK‐8 assays that 10 μM is a safe concentration, we then used 10 μM glutamate for subsequent experiments (Figure S4). At the molecular level, glutamate stimulation enhanced the expression of PGC1α and mitochondrial proteins TOMM20, indicating activation of mitochondrial biogenesis (Figure 5f,g). These were consistent with previously reports [33]. To directly assess whether glutamate stimulation restores mitochondrial function, we performed Seahorse extracellular flux analysis. Compared with untreated HTOs, FA exposure markedly reduced oxygen consumption rate and ATP production. Notably, glutamate treatment significantly preserved mitochondrial respiration and ATP output in FA‐treated HTOs, with OCR and ATP production levels comparable to those of control HTOs (Figure S4). These results indicate that glutamate stimulation maintains mitochondrial respiratory capacity and energy production under FA stress. Our data demonstrated that glutamate treatment increased overall neuronal density and structural complexity within HTOs (Figure S4). Consistently, the proportion of POMC^+^ neurons and the level of α‐MSH were significantly higher than those in FA‐treated controls (Figure 5h,i). Finally, both patch‐clamp recordings and calcium imaging confirmed that glutamate treatment restored neuronal excitability and calcium dynamics to near‐normal levels (Figures 5j and S4).

Collectively, these results demonstrate that either pharmacological activation of PGC1α or excitatory cortical‐like glutamatergic stimulation can effectively mitigate FA‐induced mitochondrial and neuronal dysfunction, supporting the notion that cortical input confers metabolic resilience through PGC1α‐dependent mitochondrial enhancement.

## 4 Discussion

In this study, we reconstructed a human CO‐HTO assembloid system to explore how cortical input modulates hypothalamic responses to lipid overload. We found that exposure to FA could selectively impair neuronal survival and activity in HTOs but not in striatal or midbrain organoids. Remarkably, cortical‐hypothalamic fusion conferred robust protection against FA‐induced apoptosis and functional decline, accompanied by preserved mitochondrial respiration and reduced lipid accumulation. Transcriptomic and functional analyses revealed that this protection might be mediated through activation of PGC1α‐dependent mitochondrial biogenesis. Furthermore, pharmacological activation of PGC1α or glutamate stimulation mimicking cortical excitatory input partially reproduced these protective effects. Together, our findings uncover a previously unrecognised mechanism by which cortical excitatory input sustains hypothalamic mitochondrial fitness through PGC1α activation, thereby conferring resilience against metabolic stress.

The hypothalamus plays the central role in energy homeostasis and lipid sensing; it was consistent with previous studies showing the degeneration of POMC^+^ neurons in high‐fat diet conditions [34, 35]. In our organoid model, FA exposure caused pronounced apoptosis, reduced α‐MSH secretion, and diminished neuronal excitability, all hallmarks of hypothalamic dysfunction observed in obesity and diabetes [36, 37]. These findings suggest that HTOs could serve as an in vitro model for assessing FA‐induced toxicity. As supported by previous evidence, cortical glutamatergic signalling has been linked to obesity‐related behaviours, likely by modulating impulsivity and food craving [11, 38]. The establishment of a CO‐HTO assembloid model allowed us to directly assess the impact of cortical input on hypothalamic function. Within this interconnected system, cortical axons extended into the hypothalamic compartment, forming functional synapses as verified by optogenetic stimulation. Remarkably, cortical fusion promoted hypothalamic neuronal maturation, enhanced excitability, and substantially mitigated FA‐induced apoptosis and α‐MSH loss. These findings indicate that cortical excitatory input can preserve hypothalamic neuronal integrity and restore mitochondrial metabolism under FA stress. Hence, our findings provided a platform to explore mechanisms of cortical regulation and potential therapeutic interventions.

Our bulk RNA‐seq and functional approaches provided molecular insight into how cortical organoids reshapes the transcriptome and stabilises mitochondrial metabolism in HTOs. PGC1α is a master regulator of mitochondrial biogenesis and oxidative metabolism, and our data showed it was robustly upregulated in fused hypothalamic tissues at both transcript and protein levels, consistent with previous reports that elevated PGC1α could drive mitochondrial expansion [30]. Correspondingly, we observed an increased number of mitochondria around lipid droplets and enhanced mitochondrial function. Moreover, exogenous glutamate treatment demonstrated that cortical excitatory input engages PGC1α expression and activates the mitochondrial programme in hypothalamic neurons. These findings were consistent with previous evidence that glutamatergic neurotransmission could modulate PGC1α and underscore a mechanistic link between cortical activity and hypothalamic metabolic resilience [33]. Furthermore, we revealed the role of glutamate in inducing PGC1α‐mediated metabolic resilience in HTO.

Whilst our study demonstrates that cortical inputs enhance the functional resistance of hypothalamic organoids to lipid overload, the underlying changes in lipid composition remain to be defined. Future lipid metabolomic analyses of this co‐culture system would be critical to directly quantify the specific lipid metabolites and pathways that mediate this protective effect, thereby bridging the gap between neuronal circuit activity and metabolic adaptation.

## 5 Conclusions

Taken together, our study elucidated that cortical organoids could improve metabolic resilience of hypothalamic organoids by modulating PGC1α‐mediated mitochondrial enhancement. We also established the CO‐HTO assembloid as a versatile platform for exploring inter‐regional brain interactions and screening potential therapeutics for metabolic disorders.

## Author Contributions

Y.L. conceived and supervised the study. M.T. and W.Z. wrote the manuscript. M.T., X.D., Q.C., and W.M. performed the experiments. S.L., M.X. and J.L. contributed to methodology and investigation. Y.G. provided advice and assistance in this study.

## Funding

This work was supported by the National Key Research and Development Programme of China (2022YFA1104800, 2021YFA1101800, 2023YFF1203600), National Natural Science Foundation of China (82325015, 82171528, 82574367, 22274079), and Natural Science Foundation of Jiangsu Province, BK20240131.

## Disclosure

All authors were provided with the full manuscript for comments and critiques before submission.

## Ethics Statement

Cell line was obtained from WiCell. All procedures were conducted in accordance with the principles outlined in the World Medical Association (WMA) Declaration of Helsinki and the Department of Health and Human Services Belmont Report.

## Conflicts of Interest

The authors declare no conflicts of interest.

## Supporting information


**Figure S1:** Cellular proliferation and viability in hypothalamic organoids, related to Figure 1.
a. Representative immunostaining of SOX2 and Ki67 in HOs at day 32, and quantification of the percentage of Ki67^+^ cells amongst SOX2^+^ cells (*N* ≥ 15 organoids from 3 replicates; mean ± SEM).
b. Representative images of fragmented cells at day 32 and quantification of their percentage (*N* ≥ 14 organoids from 3 replicates; mean ± SEM).
c. Western blot analysis of cleaved caspase‐3 expression and quantification normalised to GAPDH (*N* = 3 biological replicates; mean ± SEM, ***p < 0.001).
d. Representative calcium imaging traces from hypothalamic organoids under different conditions and quantification of peak [Ca^2+^] changes ((Fmax–F0)/F0; *N* ≥ 22 cells; mean ± SEM, ***p < 0.001).
e. qPCR analysis of CD36 and FFAR1 expression in D20 and D40 HTO organoids. Mean ± SEM, **p < 0.01, ***p < 0.001.

**Figure S2:** Comparative characterisation of striatal and midbrain organoids, related to Figure 1.
a. Representative immunostaining of GSX2 and TUJ1 in striatal organoids at day 32, and quantification of fragmented cells (*N* ≥ 15 organoids from 3 replicates; mean ± SEM).
b. Representative images of FOXA2 and NESTIN in midbrain organoids at day 32, and quantification of fragmented cells (*N* ≥ 13 organoids from 3 replicates; mean ± SEM).
c. Representative traces of action potentials recorded from neurons within striatal organoids and quantification of the amplitude of the first evoked AP (*N* ≥ 6 organoids from 2 replicates; mean ± SEM).
d. Representative traces of action potentials recorded from neurons within midbrain organoids and quantification of the amplitude of the first evoked AP (*N* ≥ 8 organoids from 2 replicates; mean ± SEM).
e. Expression levels of CD36, FFAR1, and FATP4 at day 32 determined by qPCR (*N* = 3 biological replicates; mean ± SEM).

**Figure S3:** Characterisation of cortical–hypothalamic assembloids, related to Figure 2.
a. Schematic illustration of the protocol for generating cortical organoids from human iPSCs. Confocal images showing immunostaining for FOXG1, TUJ1, CTIP2, DCX, TBR1, MAP2, and Hoechst at day 30. Quantification of PAX6^+^/CTIP2^+^ cells in cortical organoids at day 30 (*N* ≥ 12 organoids from 3 independent experiments). Confocal images showing immunostaining for SOX2, Ki67, and TUJ1 in cortical organoids at day 30, and for SATB2 and DCX at day 60.
b. Bright‐field images of cortical‐hypothalamic assembloids at different fusion stages and quantification of their diameters.
c. Confocal images of GFP immunostaining at 2 days post fusion. Bright‐field images of assembloids in which cortical organoids were infected with ChR2.
d. Representative images of GFP^+^ neurons projecting into hypothalamic organoids at day 40, and quantification of neurite complexity, including branch number and the length of the longest neurite (*N* ≥ 21 neurons from 3 replicates; data are presented as mean ± SEM; *p < 0.05, ***p < 0.001).
e. Representative traces of sodium and potassium currents recorded at day 40.
f. Schematic illustration of the protocol for infecting cortical organoids with mCherry followed by fusion with hypothalamic organoids. Representative calcium imaging traces from hypothalamic organoids within assembloids under different conditions, and quantification of peak [Ca^2+^] changes ((Fmax–F0)/F0; *N* ≥ 25 cells; mean ± SEM).

**Figure S4:** Quantitative analyses of mitochondrial function and neuronal activity, related to Figure 5.
a. qPCR analysis of PGC1α expression in HTO organoids. Mean ± SEM, ****p < 0.0001.
b. Quantification of basal respiration and ATP production at D42 across different groups (*p < 0.05, **p < 0.01; mean ± SEM).
c. Quantification of fragmented cells at D42 (**p < 0.01; mean ± SEM).
d. Representative calcium imaging in hypothalamic organoids from different groups and quantification of peak [Ca^2+^] changes ((Fmax–F0)/F0; *N* ≥ 25 cells; *p < 0.05, ***p < 0.001; mean ± SEM).
e. CCK‐8 assay assessing cell viability following various concentrations of L‐glutamate treatment (***p < 0.0001, one‐way ANOVA; mean ± SEM).
f. Mitochondrial oxygen consumption rate measured using a Seahorse XF96 Analyser and quantification of ATP production at D42 HTO and HTO + Glu groups. (*p < 0.05, ***p < 0.001; mean ± SEM).
g. Mitochondrial oxygen consumption rate measured using a Seahorse XF96 Analyser.
Quantification of ATP production at D42 HTO, HTO + FA and HTO + Glu + FA groups. (*p < 0.05, ***p < 0.001; mean ± SEM).
h. Representative images of GFP^+^ neurons within hypothalamic organoids from different groups and quantification of neurite branch number (*p < 0.01; mean ± SEM).
i. Calcium imaging traces of hypothalamic organoids at different stages across groups and quantification of peak [Ca^2+^] changes ((Fmax‐F0)/F0; *N* ≥ 25 cells; **p < 0.01, ***p < 0.001; mean ± SEM).

**Table S1:** The list of key reagents and antibodies.

## Data Availability

The data that support the findings of this study are openly available in Genome Sequence Archive for Human at https://ngdc.cncb.ac.cn/search/specific?db=hra&q=HRA014636, reference number HRA014636.
