# Supplementary material for: Cortical‐Hypothalamic Assembloids Uncover the Cortical Regulation of Hypothalamic Responses to Fatty Acid
Source: Cell Prolif. 2026 Apr 6;59(8):e70207. doi: 10.1111/cpr.70207 (PMC13325828; doi:10.1111/cpr.70207)
Supplement: Supplementary file 1 [file CPR-59-e70207-s001.docx]

| **Reagent Name** | **Source（Cat. No）** |
| --- | --- |
| Vitronectin | Thermos, A31804 |
| Essential 8 | Gibico, A1517001 |
| Dispase | Gibico, 17105041 |
| N2 supplement | Gibico, 17502048 |
| non-essential amino acids (NEAA) | Gibico, 11140 |
| Dulbecco's Modified Eagle Medium/Nutrient Mixture F-12(DMEM/F12) | Gibico, 11320033 |
| SB-431542 | Tocris, 1614 |
| DMH1 | Tocris, 4126 |
| IWR-1 | Tragetmol, T2651 |
| Fetal bovine serum (FBS) | Gibico, 10099-141 |
| B-27 Supplement | Gibico, 12587010 |
| A83-01 | StemCell Technologies, 72024 |
| SAG | Sigma, 566660-5MGCN |
| Purmorphamine | Tocris, 455110 |
| SHH C25II | R&D Systems, 464-SH |
| BDNF | Gibco, 450-02-100UG |
| GDNF | Gibco, AF-450-10-100 |
| cAMP | Sigma, D0260 |
| Ascorbic acid | Sigma, 1043003-1G |
| GlutaMAX | Gibco, 35050061 |
| SHH C24II | R&D Systems,1845-SH |
| CHIR99021 | Stemgent, 040004 |
| FGF8b | Novoprotein, C798 |
| Donkey serum | Sigma, S30-M |
| Paraformaldehyde (PFA) | Sigma, 158127 |
| Triton X-100 | Beyotime, P0096 |
| Fluo-4, AM | Thermos, F14201 |
| Cell Counting Kit-8 | Beyotime, C0037 |
| SDS-PAGE protein loading buffer | Beyotime, TBSTP0015 |
| Cocktail | Sigma, 11697498001 |
| TBST Buffer | Sangon Biotech, C520009-0001 |
| TRIzol kit | Thermo, 15596026 |
| PrimeScript RT reagent Kit (Perfect Real Time) | Takara, RR037Q |
| Penicillin-streptomycin (PS) | Gibco, 2441849 |

| **Antibody** | **Source** | **Cat. No** |
| --- | --- | --- |
| NKX2.1 | Santa | SC-13040 |
| ISL1 | DSHB | 40.2D6 |
| NESTIN | Santa | SC-21247 |
| TBX3 | Absmart | T58034 |
| RAX | Santa | SC-376837 |
| OTP | Absmart | ME025855S |
| MAP2 | Sigma | M1406 |
| POMC | HUABIO | JE32-88 |
| PAX6 | DSHB | PAX6 |
| FOXG1 | Abcam | AB18259 |
| CTIP2 | Abcam | AB18465 |
| SATB2 | Abcam | AB51502 |
| DCX | Cell Signaling Technology | 4604 |
| TUJ1 | Sigma | T8660 |
| GFP | Abcam | AB3080 |
| GSH2 | Millipore | ABN162 |
| FOXA2 | Santa | SC-374376 |
| NEUN | Arigo | ABG52283 |
| KI67 | Thermo Fisher Scientific | 180191z |
| SOX2 | R&D Systems | AF2018 |
| 488 Donkey Anti-Goat | Thermo Fisher Scientific | A11055 |
| 488 Donkey Anti-Mouse | Thermo Fisher Scientific | A21202 |
| 488 Donkey Anti-Rabbit | Thermo Fisher Scientific | A21206 |
| 546 Donkey Anti-Goat | Thermo Fisher Scientific | A11056 |
| 546 Donkey Anti-Mouse | Thermo Fisher Scientific | A10036 |
| 546 Donkey Anti-Rabbit | Thermo Fisher Scientific | A10040 |
| Hoechst33258 | Thermo Fisher Scientific | H1399 |
