# Supplementary figures and images for: Cortical‐Hypothalamic Assembloids Uncover the Cortical Regulation of Hypothalamic Responses to Fatty Acid
Source: Cell Prolif. 2026 Apr 6:e70207. Online ahead of print. doi: 10.1111/cpr.70207 (PMC13325828; doi:10.1111/cpr.70207)

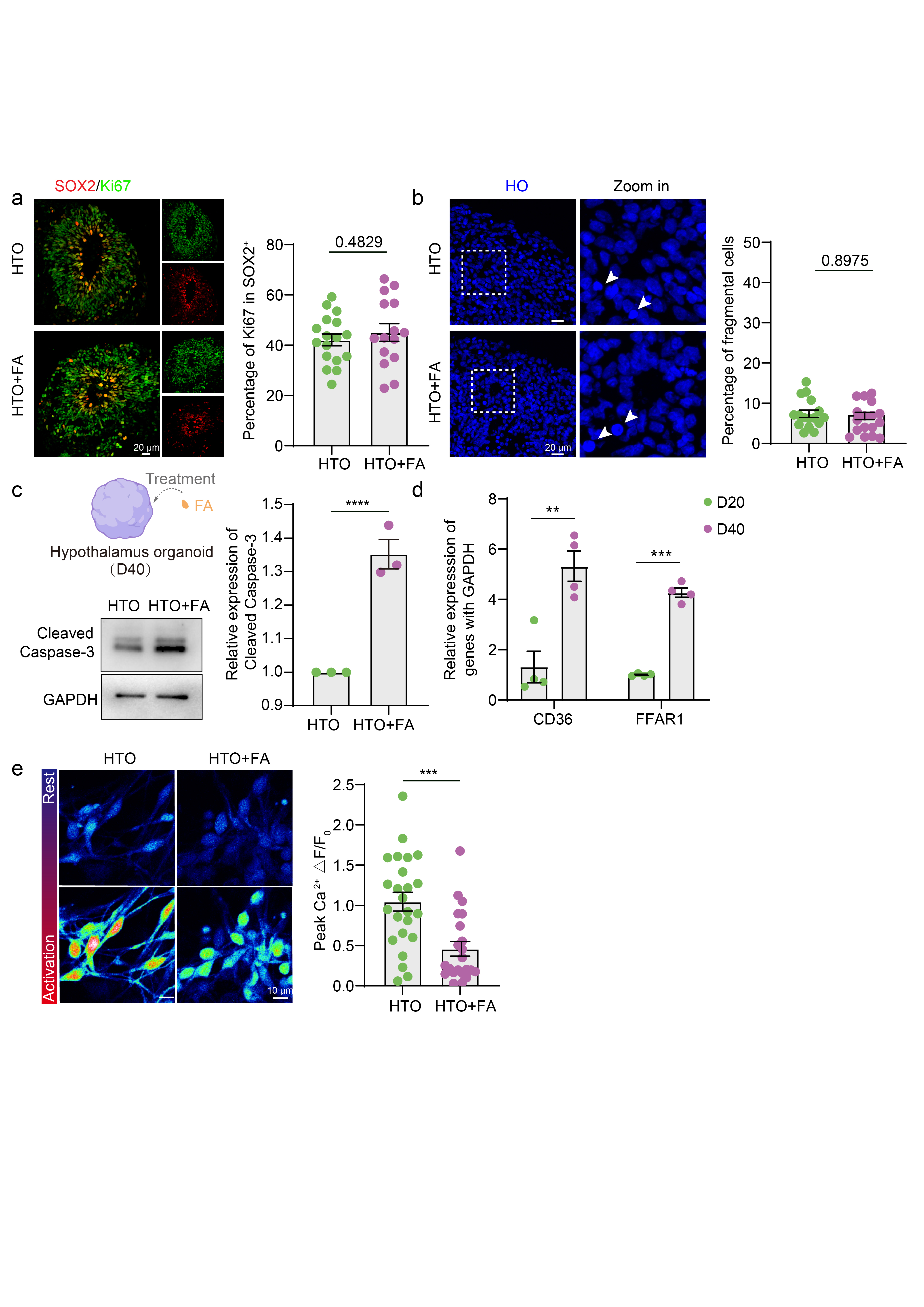

Supplement: Supplementary file 1 — Figure S1: Cellular proliferation and viability in hypothalamic organoids, related to Figure 1. a. Representative immunostaining of SOX2 and Ki67 in HOs at day 32, and quantification of the percentage of Ki67+ cells amongst SOX2+ cells (N ≥ 15 organoids from 3 replicates; mean ± SEM). b. Representative images of fragmented cells at day 32 and quantification of their percentage (N ≥ 14 organoids from 3 replicates; mean ± SEM). c. Western blot analysis of cleaved caspase‐3 expression and quantification normalised to GAPDH (N = 3 biological replicates; mean ± SEM, ***p < 0.001). d. Representative calcium imaging traces from hypothalamic organoids under different conditions and quantification of peak [Ca2+] changes ((Fmax–F0)/F0; N ≥ 22 cells; mean ± SEM, ***p < 0.001). e. qPCR analysis of CD36 and FFAR1 expression in D20 and D40 HTO organoids. Mean ± SEM, **p < 0.01, ***p < 0.001. [file CPR-9999-e70207-s002.jpg]

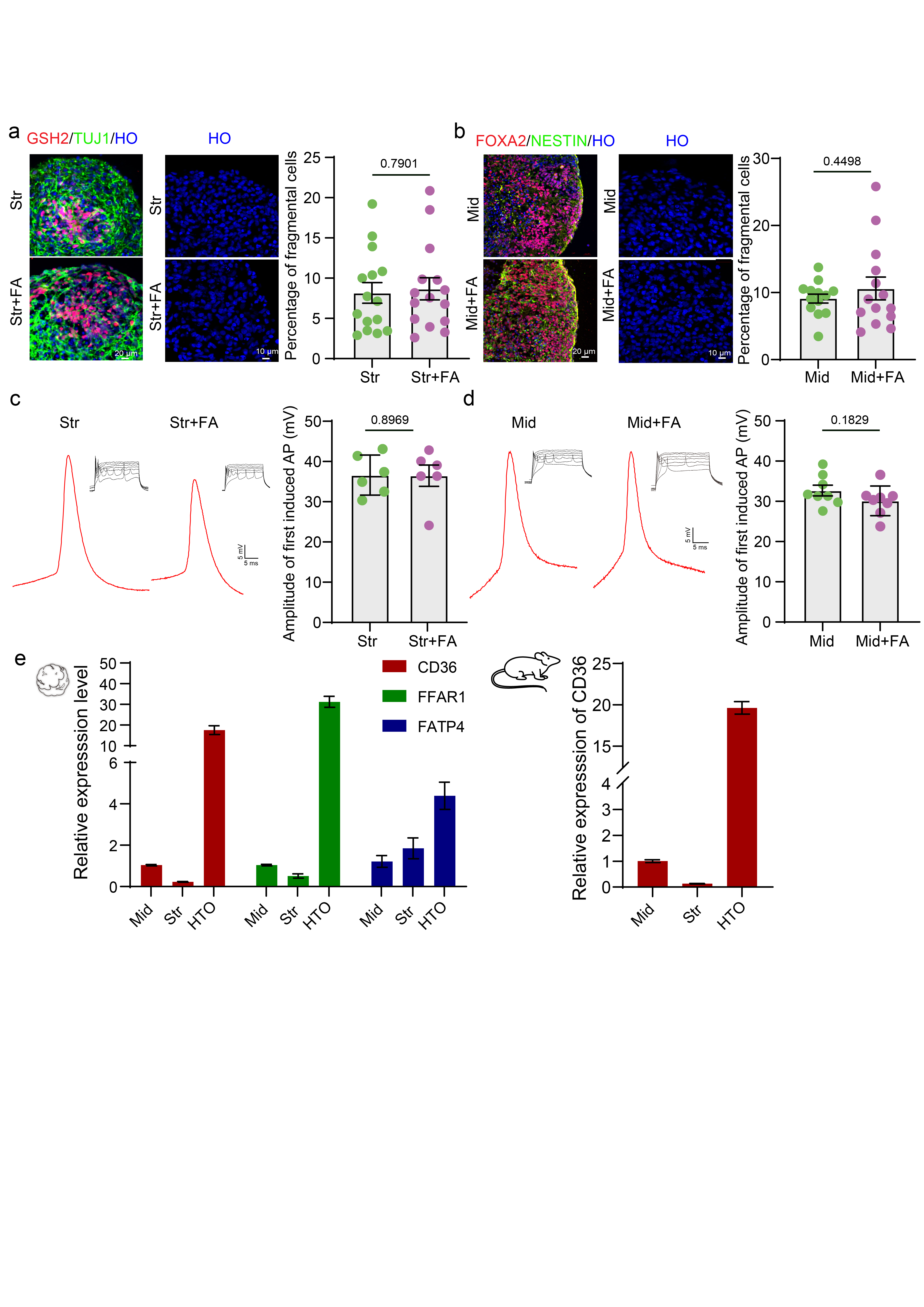

Supplement: Supplementary file 2 — Figure S2: Comparative characterisation of striatal and midbrain organoids, related to Figure 1. a. Representative immunostaining of GSX2 and TUJ1 in striatal organoids at day 32, and quantification of fragmented cells (N ≥ 15 organoids from 3 replicates; mean ± SEM). b. Representative images of FOXA2 and NESTIN in midbrain organoids at day 32, and quantification of fragmented cells (N ≥ 13 organoids from 3 replicates; mean ± SEM). c. Representative traces of action potentials recorded from neurons within striatal organoids and quantification of the amplitude of the first evoked AP (N ≥ 6 organoids from 2 replicates; mean ± SEM). d. Representative traces of action potentials recorded from neurons within midbrain organoids and quantification of the amplitude of the first evoked AP (N ≥ 8 organoids from 2 replicates; mean ± SEM). e. Expression levels of CD36, FFAR1, and FATP4 at day 32 determined by qPCR (N = 3 biological replicates; mean ± SEM). [file CPR-9999-e70207-s001.jpg]

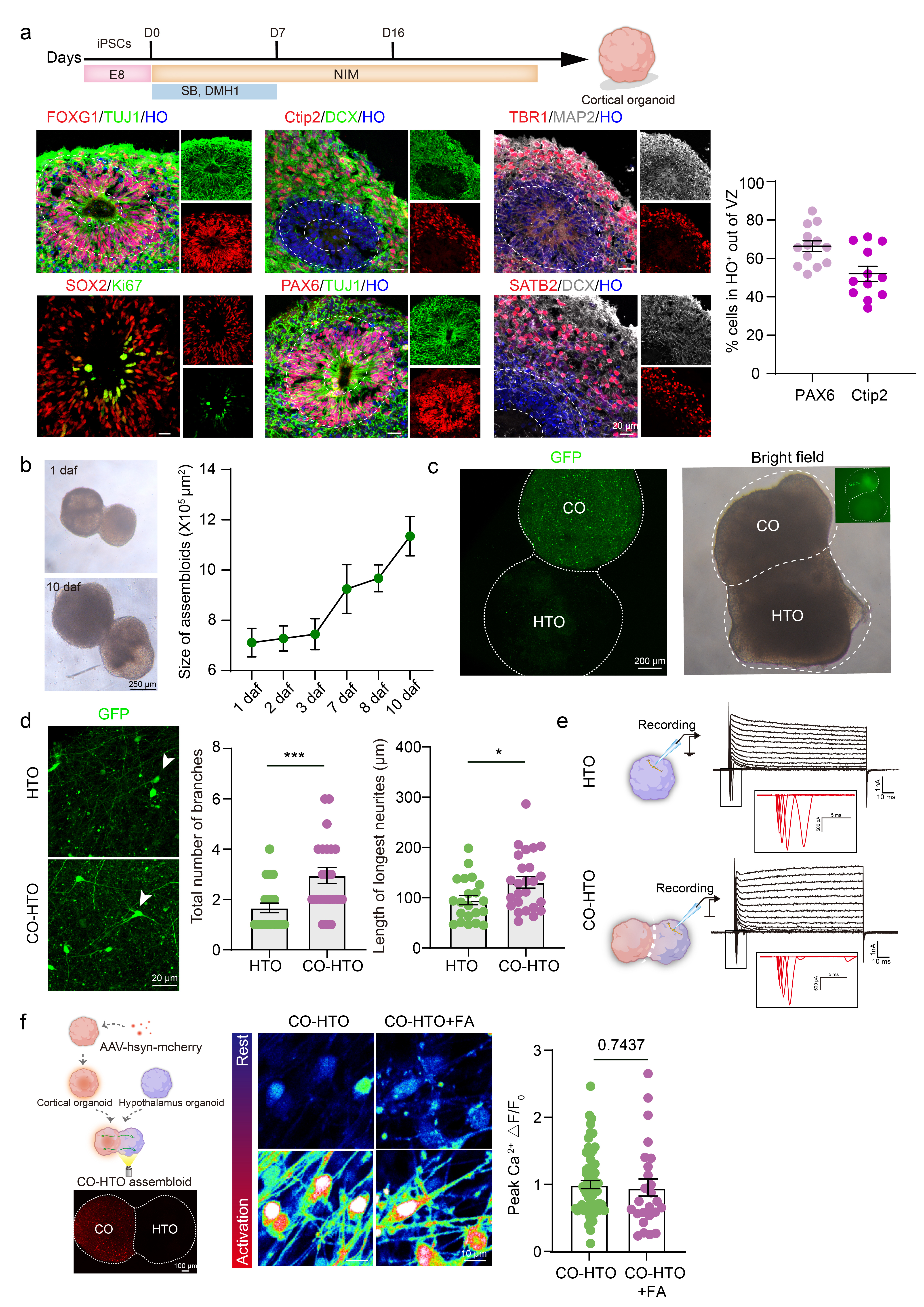

Supplement: Supplementary file 3 — Figure S3: Characterisation of cortical–hypothalamic assembloids, related to Figure 2. a. Schematic illustration of the protocol for generating cortical organoids from human iPSCs. Confocal images showing immunostaining for FOXG1, TUJ1, CTIP2, DCX, TBR1, MAP2, and Hoechst at day 30. Quantification of PAX6+/CTIP2+ cells in cortical organoids at day 30 (N ≥ 12 organoids from 3 independent experiments). Confocal images showing immunostaining for SOX2, Ki67, and TUJ1 in cortical organoids at day 30, and for SATB2 and DCX at day 60. b. Bright‐field images of cortical‐hypothalamic assembloids at different fusion stages and quantification of their diameters. c. Confocal images of GFP immunostaining at 2 days post fusion. Bright‐field images of assembloids in which cortical organoids were infected with ChR2. d. Representative images of GFP+ neurons projecting into hypothalamic organoids at day 40, and quantification of neurite complexity, including branch number and the length of the longest neurite (N ≥ 21 neurons from 3 replicates; data are presented as mean ± SEM; *p < 0.05, ***p < 0.001). e. Representative traces of sodium and potassium currents recorded at day 40. f. Schematic illustration of the protocol for infecting cortical organoids with mCherry followed by fusion with hypothalamic organoids. Representative calcium imaging traces from hypothalamic organoids within assembloids under different conditions, and quantification of peak [Ca2+] changes ((Fmax–F0)/F0; N ≥ 25 cells; mean ± SEM). [file CPR-9999-e70207-s005.jpg]

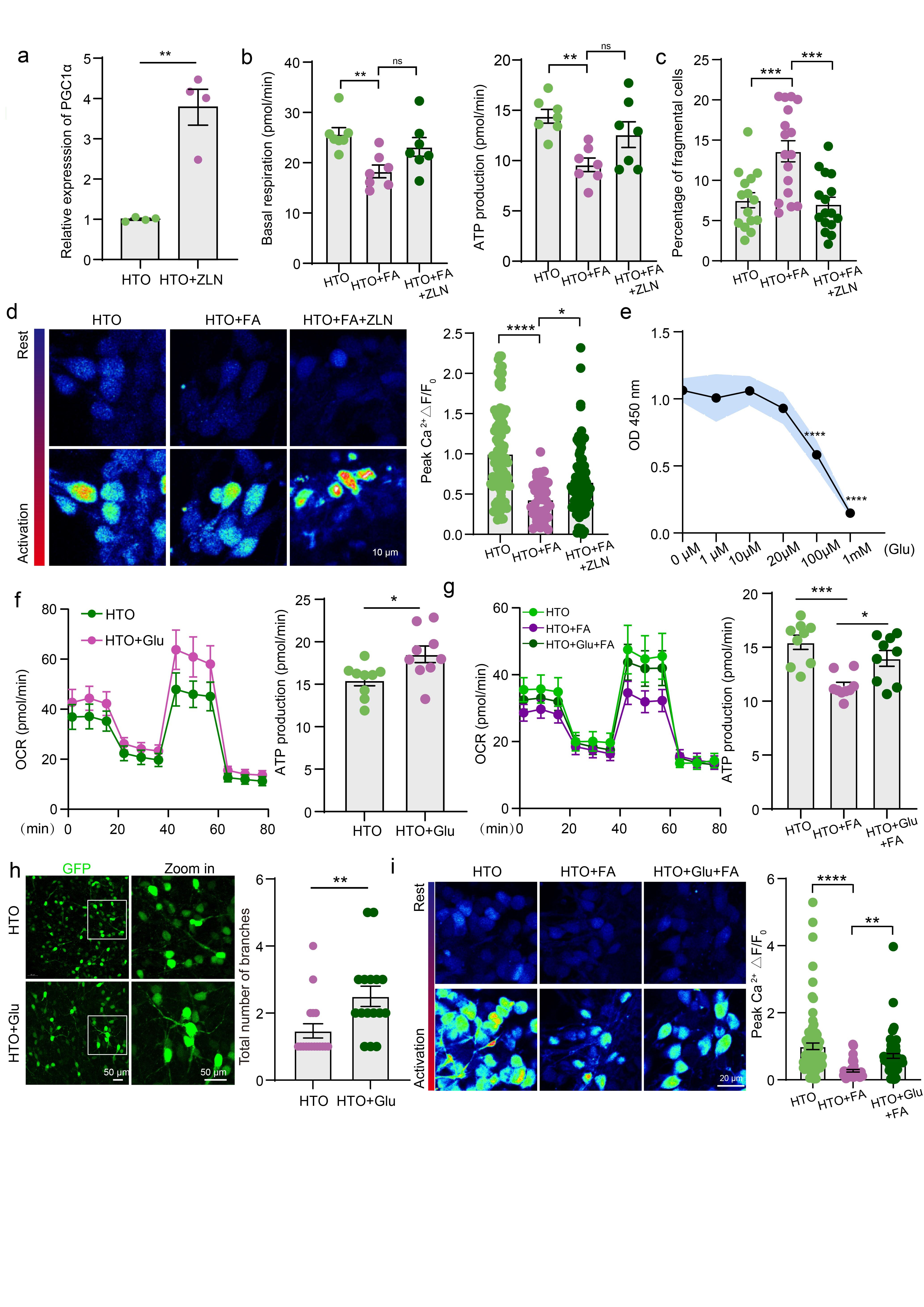

Supplement: Supplementary file 4 — Figure S4: Quantitative analyses of mitochondrial function and neuronal activity, related to Figure 5. a. qPCR analysis of PGC1α expression in HTO organoids. Mean ± SEM, ****p < 0.0001. b. Quantification of basal respiration and ATP production at D42 across different groups (*p < 0.05, **p < 0.01; mean ± SEM). c. Quantification of fragmented cells at D42 (**p < 0.01; mean ± SEM). d. Representative calcium imaging in hypothalamic organoids from different groups and quantification of peak [Ca2+] changes ((Fmax–F0)/F0; N ≥ 25 cells; *p < 0.05, ***p < 0.001; mean ± SEM). e. CCK‐8 assay assessing cell viability following various concentrations of L‐glutamate treatment (***p < 0.0001, one‐way ANOVA; mean ± SEM). f. Mitochondrial oxygen consumption rate measured using a Seahorse XF96 Analyser and quantification of ATP production at D42 HTO and HTO + Glu groups. (*p < 0.05, ***p < 0.001; mean ± SEM). g. Mitochondrial oxygen consumption rate measured using a Seahorse XF96 Analyser. Quantification of ATP production at D42 HTO, HTO + FA and HTO + Glu + FA groups. (*p < 0.05, ***p < 0.001; mean ± SEM). h. Representative images of GFP+ neurons within hypothalamic organoids from different groups and quantification of neurite branch number (*p < 0.01; mean ± SEM). i. Calcium imaging traces of hypothalamic organoids at different stages across groups and quantification of peak [Ca2+] changes ((Fmax‐F0)/F0; N ≥ 25 cells; **p < 0.01, ***p < 0.001; mean ± SEM). [file CPR-9999-e70207-s003.jpg]
